# Brain microabscesses in a porcine model of *Staphylococcus aureus* sepsis

**DOI:** 10.1186/1751-0147-55-76

**Published:** 2013-10-31

**Authors:** Lærke B Astrup, Mette V Nielsen, Tine M Iburg, Páll S Leifsson, Henrik E Jensen, Ole L Nielsen, Jørgen S Agerholm

**Affiliations:** 1 Section of Experimental Animal Models, Department of Veterinary Disease Biology, Faculty of Health and Medical Sciences, University of Copenhagen, Ridebanevej 3, DK-1870, Frederiksberg C, Denmark; 2 Department of Pathology and Wildlife Diseases, National Veterinary Institute, SE-751 89, Uppsala, Sweden; 3 Department of Large Animal Sciences, Faculty of Health and Medical Sciences, University of Copenhagen, Dyrlægevej 68, DK-1870, Frederiksberg C, Denmark

**Keywords:** Pig, *Staphylococcus aureus*, Brain, Model, Sepsis, Abscess, Haematogenous

## Abstract

**Background:**

Sepsis caused by *Staphylococcus aureus* often leads to brain microabscesses in humans. Animal models of haematogenous brain abscesses would be useful to study this condition in detail. Recently, we developed a model of *S. aureus* sepsis in pigs and here we report that brain microabscesses develop in pigs with such induced *S. aureus* sepsis.

Twelve pigs were divided into three groups. Nine pigs received an intravenous inoculation of *S. aureus* once at time 0 h (group 1) or twice at time 0 h and 12 h (groups 2 and 3). In each group the fourth pig served as control. The pigs were euthanized at time 12 h (Group 1), 24 h (Group 2) and 48 h (Group 3) after the first inoculation. The brains were collected and examined histopathologically.

**Results:**

All inoculated pigs developed sepsis and seven out of nine pigs developed brain microabscesses. The microabscesses contained *S. aureus* and were located in the prosencephalon and mesencephalon. Chorioditis and meningitis occurred from 12 h after inoculation.

**Conclusions:**

Pigs with experimental *S. aureus* sepsis often develop brain microabscesses. The porcine brain pathology mirrors the findings in human sepsis patients. We therefore suggest the pig as a useful animal model of the development of brain microabscesses caused by *S. aureus* sepsis.

## Background

*Staphylococcus aureus* is a leading cause of sepsis in humans [1]. Autopsy studies have revealed a high frequency of microabscesses in the brain of sepsis patients. The occurrence of brain microabscesses is especially high in sepsis caused by *S. aureus*[2]. Despite this, brain involvement is often an unrecognized complication in sepsis patients. This is because of several diagnostic difficulties: the symptoms of brain microabscesses can be vague and fleeting, and such symptoms can be difficult to distinguish in gravely ill sepsis patients [2]. Therefore, brain microabscesses are prone to diagnostic delay. The consequence of this is an immense lack of knowledge about the course and pathology of sepsis-related microabscesses in the brain.

Recently, we developed a porcine model of sepsis based on intravenous inoculation of *S. aureus*[3]. We used this model to study systemic effects of sepsis but examination of brain lesions was not carried out [3]. However, the neurological complications in humans with *S. aureus* sepsis make the brain a highly relevant organ to evaluate in models of sepsis. Therefore, we undertook this study to evaluate if pigs develop brain microabscesses during experimental *S. aureus* sepsis.

## Methods

Brain tissue from 12 female SPF Yorkshire/Landrace pigs with a BW of 20–25 kg from an earlier study of sepsis were used [3]. The pigs were acclimatized for 7 days prior to the trial and were fasted for the last 12 h prior to inoculation. The pigs were clinically examined immediately before the trial and randomly assigned into 3 groups (1–3). The pigs were sedated and anaesthetised before and during bacterial inoculation. Sedation was achieved by intramuscular injection with a solution containing zolazepam, tiletamine, xylazine and ketamine, (0.83 mg/kg BW of each drug), and buthorphanol (0.17 mg/kg BW). A 22G catheter was inserted into the right ear vein for infusion of anaesthetics. Anaesthesia was achieved with a solution containing xylazine (1 mg/ml), ketamine (2 mg/ml), buthorphanol (0.1 mg/ml), and guaifenesine (48 mg/ml). Anaesthetics were administrated as dose-to-effect and only during inoculation. A 22G catheter was inserted into the left ear vein for inoculation of *S. aureus* strain S54F9 (*spa* type t1333) [4,5]*.* Nine pigs received a saline suspension of 10^8^ colony forming units *S. aureus* ml/kg BW [4]. The suspension was administrated once at 0 h (group1) or twice at 0 h and 12 h (groups 2 and 3). In each group one pig served as control and received mock inoculation(s) with sterile saline. The sedation, insertion of ear-vein catheters, and anaesthetization were repeated during subsequent inoculations. At termination of the trial, the pigs were sedated with an intramuscular injection with a solution containing zolazepam, tiletamine, xylazine and ketamine, (0.83 mg/kg BW of each drug), and buthorphanol (0.17 mg/kg BW). After sedation the pigs were euthanized with pentobarbital intracardially. Pigs were euthanized at 12 h (Group 1), 24 h (Group 2) and 48 h (Group 3) after the first inoculation with bacteria (Table 1). During the entire trial pigs were closely monitored and evaluated on appearance and general condition. Any sign of severe pain would have prompted immediate euthanasia as stated in our protocol for human endpoints [3]. As such, the animal welfare concerns related to sepsis models were managed by several different aspects of the study design; the maximum time frame of 48 h, the human endpoints, the close monitoring of the animals, and the low number of experimental animals. The entire procedure was approved by the Danish Animal Experimental Act (licence No. 2008/561-1462).

**Table 1:** **Experimental design and pathological changes in *Staphylococcus aureus *** **inoculated pigs**

**Group no.**	**Pig no.**	**Inoculation**	**Euthanisia**	**Number of brain microabscesses**	**Suppurative meningitis**	**Suppurative choroiditis**
1	1	0 h	12 h	4	0	0
	2	0 h	12 h	8	+	0
	3	0 h	12 h	0	+	+
	4 (control)	0 h, mock	12 h	0	0	0
2	5	0 h + 12 h	24 h	3	+	+
	6	0 h + 12 h	24 h	10	++	+
	7	0 h + 12 h	24 h	2	++	+
	8 (control)	0 h + 12 h, mock	24 h	0	0	0
3	9	0 h + 12 h	48 h	1	+++	+++
	10	0 h + 12 h	48 h	0	+	+
	11	0 h + 12 h	48 h	3	+++	+++
	12 (control)	0 h + 12 h, mock	48 h	0	0	0

In the nervous tissue the number of microabscesses was counted and summed up for each animal. The infiltration of neutrophils in the meninges and in the choroid plexus was scored on a semi quantitative scale. The semi quantitative score of each pig was found by this method: Ten fields with the tissue of interest were randomly sampled in each section from each pig. The fields were examined at 40× objective. The number of neutrophils in the tissue of interest were counted in each field and summed up for all sections to yield a total number for each pig. Based on this total number of neutrophils each pig was then scored on the following scale: 0 = ≤ 15; + = 16 – 50; ++ = 51 – 100; +++ = ≥100.

The pigs were necropsied immediately after euthanasia. For use in this study-part brains were sampled and fixed by immersion in 10% neutral buffered formalin. After fixation the brains were divided through the longitudinal cerebral fissure. One brain-half was randomly selected from each pig and cut in 4 mm thick coronal slabs [6]. From each coronal slab, a section of 4 μm was cut and stained with haematoxylin and eosin. Additional sections were subjected to immunohistochemistry to visualize *S. aureus* antigen according to a previously developed protocol [7]. Each section was examined for inflammatory reactions in the three tissues: the nervous tissue, the choroid plexus, and the meninges. In the nervous tissue the abscesses were counted. In the choroid plexus and in the meninges the number of neutrophils was scored on a semi-quantitative scale (Table 1). The histological examination was done blinded.

## Results

All infected pigs developed sepsis as reported earlier [3]. Necropsy revealed no macroscopic changes in the brains.

**Figure 1:**
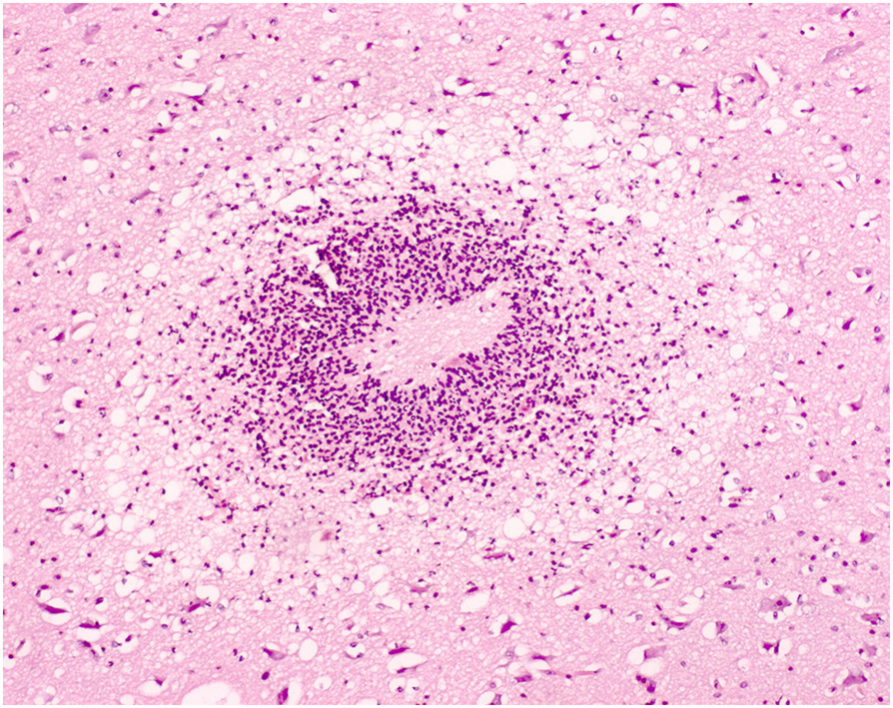
**Focal brain microabscess.** The abscess consists of a necrotic centre surrounded by a rim of neutrophils and edema. Pig No. 2. Haematoxylin and eosin. Bar = 100 μm.

**Figure 2:**
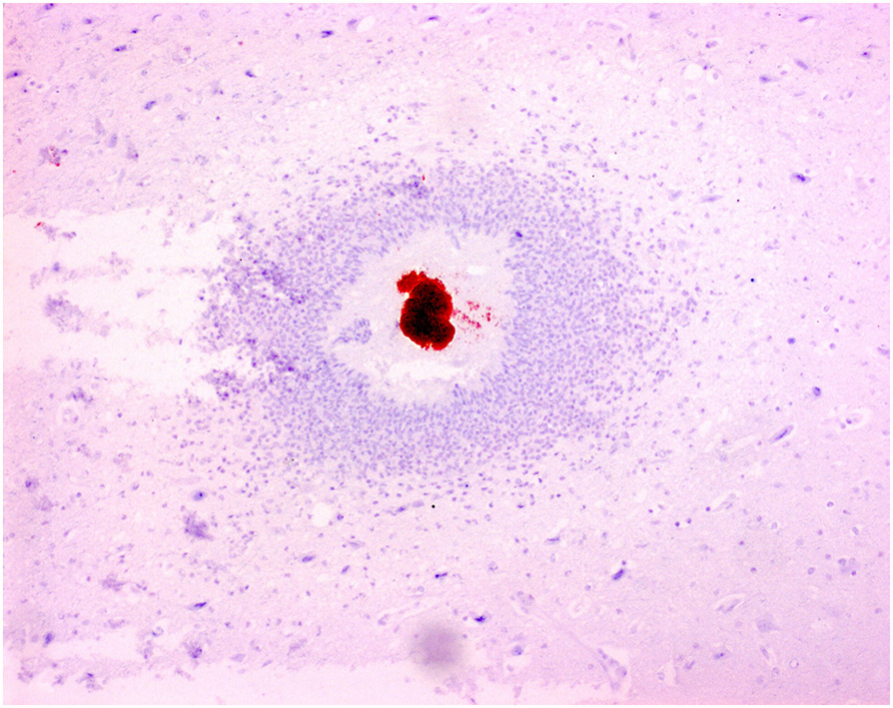
**Focal brain microabscess with *Staphylococcus aureus*.** This staining shows presence of *S. aureus* antigen in the centre of a microabscess. Pig No. 2; same abscess as in Figure 1. Immunohistochemical staining for *S. aureus* antigen. Bar = 100 μm.

Histology revealed microabscesses in the brain of seven out of nine inoculated pigs. Microabscesses occurred in all groups (1–3). All microabscesses were acute with infiltration of predominantly neutrophils and were surrounded by vacuolated neuropil (Figure 1). Immunohistochemistry showed presence of *S. aureus* antigen in the microabscesses (Figure 2). The microabscesses had a similar morphology across pigs. However, the number of microabscesses varied considerably between pigs within the same group (Table 1). All microabscesses were located in prosencephalon and mesencephalon, primarily within the territory described to be vascularized by the medial cerebral artery [8]. Out of 27 microabscesses in total, 25 were located in the grey matter and 2 were located in the white matter. In addition to presence of microabscesses, the nervous tissue displayed oedema and perivascular cuffing dominated by neutrophils (Table 1).

**Figure 3:**
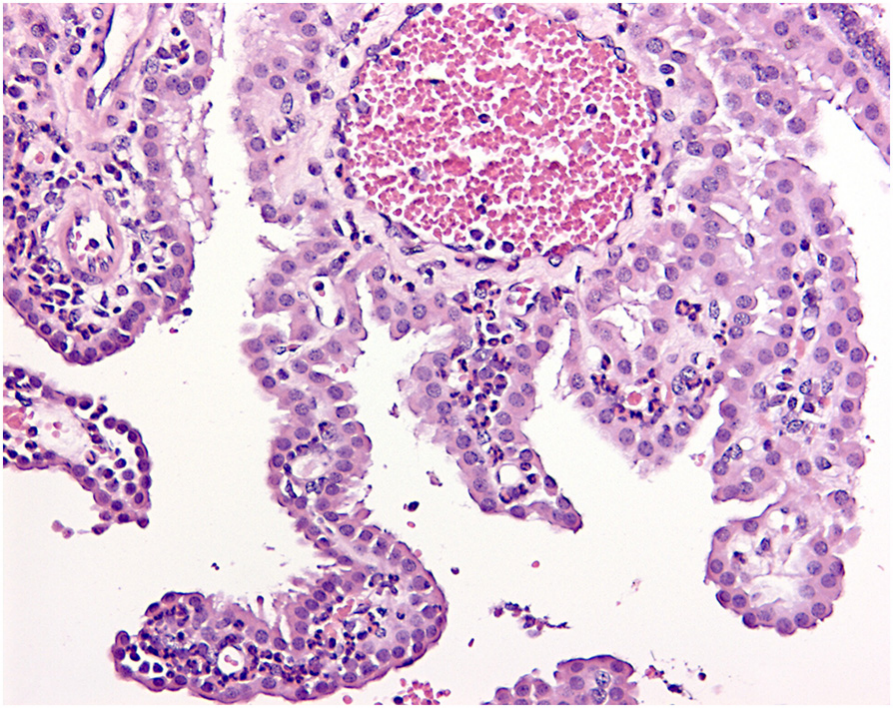
**Suppurative choroiditis.** Infiltration with neutrophils in the stroma of the choroid villi. Pig No. 5. Haematoxylin and eosin. Bar = 50 μm.

Histology of the choroid plexus revealed neutrophilic chorioditis in seven out of nine inoculated pigs (Table 1 and Figure 3). Chorioditis was occasionally accompanied by periventricular oedema and/or ependymal desquamation.

Histology of the meninges showed a multifocal occurrence of an inflammatory infiltrate in all but one inoculated pigs. This infiltrate consisted of neutrophils and/or macrophage-like cells with the neutrophils dominating until 24 h after inoculation (Table 1).

None of the control pigs showed any brain pathology.

## Discussion

Autopsy studies have revealed a high frequency of brain microabscesses in human sepsis patients. Brain microabscesses are especially frequent in sepsis caused by *S. aureus*[2]. In our study of porcine *S. aureus* sepsis, we likewise found a high frequency of brain microabscesses. The brain microabscesses occurred concurrently with inflammation in the meninges and in the choroid plexus. The occurrence of the brain microabscesses was not restricted to areas close to the meninges or the ventricular tissues. Rather, the microabscesses were primarily found within the territory of the medial cerebral artery. Hence the bacteria were probably spread from the blood to the three tissues simultaneously and not as a secondary spread from the meninges and the choroid plexus to the nervous tissue or *vice versa*. The exact mechanism(s) behind this direct colonisation of the nervous tissue with bacteria from the blood is uncertain. However, it is known that damage to the nervous tissue and the blood–brain barrier is a prerequisite for most bacterial invasions from the blood to the brain [9]. In sepsis patients such damage may be caused by several events including: immunologic and toxic damage caused by cytokines released during sepsis; haemorrhage/leakage from the vessels caused by vasculitis; or by cerebral infarction caused by either vasculitis, by disseminated intravascular coagulation, or by emboli. Emboli might even be septic and thereby cause a direct implantation of infectious material to the damaged brain area(s) [9,10]. Several of these events might also take place concurrently in a sepsis patient. Thus there can be many pathways for the development of brain abscesses in sepsis. All of these pathways seem possible in both human and porcine sepsis with one exception: different from humans pigs have a microarteriolar meshwork, the rete mirabile, interposed between the internal carotid artery and the Circle of Willis [11]. The rete mirabile probably prevents the spread of large emboli to the porcine brain [12]. This difference was however considered of minor importance to our model. The reason for this was that large human brain emboli would probably lead to severe symptoms and uni-focal abscess formation. Contrary to this, human brain abscesses in sepsis patients are described as multiple and microscopic. Furthermore, these microabscesses are often clinically overlooked [2]. As such, large emboli seem a rather rare cause of human brain abscesses in sepsis. Therefore we assumed that the capture of large emboli in rete mirabile does not compromise a porcine model of sepsis-associated brain abscesses in humans. This assumption was confirmed by our findings as we showed the same brain pathology in porcine sepsis as described in human sepsis despite the presence of a rete mirabile in the pig. This leads us to suggest the pig as an appropriate animal for models of haematogenous brain abscesses caused by sepsis. Furthermore, we consider a porcine model of haematogenous brain abscesses an important contribution to existing models of brain abscesses. Many existing models of brain abscesses use local trans-cranial induction of brain infection. Such models also often include the use of foreign materials such as agarose beads as a vehicle for the bacteria [13,14]. However, a model based on haematogenous spread of bacteria to the brain is needed to study the development of brain abscesses during sepsis. In addition, a model of haematogenous brain abscesses should preferable be developed in pigs because: pigs share many metabolic, physiological, and anatomical characteristics with humans [15]; *S. aureus* often causes metastatic infections in both pigs and humans [16,17]; brain microabscesses commonly occur in both pigs and humans with systemic bacterial infections e.g. in endocarditis [6,18]; and pigs develop sepsis after intravenous inoculation of *S. aureus*[3]. As such the rete mirabile was the only difference between humans and pigs hitherto considered an important hindrance in porcine models of haematogenous brain abscesses. This hindrance we hereby questioned. Future models of brain abscesses in sepsis might however take into consideration the possible benefits of refining our model into a local haematogenous brain model. One of the benefits of such refinement might be to spare the animals some of the symptoms related to sepsis and thus increase the animal welfare in these studies.

Altogether, we have illustrated that brain abscesses develop in porcine sepsis comparably to human sepsis pathology. We therefore suggest the pig as an appropriate animal model of human brain abscesses in sepsis despite the presence of a rete mirabile in the pig.

## Conclusions

Our study demonstrates that experimental porcine *S. aureus* sepsis causes multiple microabscesses in the brain and an inflammatory reaction in meninges and the ventricles within 12 h of infection. As such, porcine sepsis mirrors human sepsis with regard to brain pathology despite the presence of a rete mirabile in the pig. We therefore suggest the pig as a useful animal model of the development of brain microabscesses caused by *S. aureus* sepsis.

## Abbreviations

BW: Body weight; SPF: Specific pathogen free.

## Competing interests

JSA is editor-in-chief of Acta Veterinaria Scandinavia, but has not in any way been involved in or interacted with the review process or editorial decision making. The authors declare they have no competing interests.

## Authors’ contributions

TMI, PSL, HEJ, OLN and JSA conceived of the study, participated in its design and coordination, conducted the study and carried out the practical data collection. LBA carried out the histopathological and immunocytochemistry studies and drafted the manuscript. MVN contributed to the histopathological and immunocytochemistry studies and helped to draft the manuscript. PSL and JSA supervised the drafting of the manuscript. All authors read and approved the final manuscript.
